# Anatomical landmark detection on bi-planar radiographs for predicting spinopelvic parameters

**DOI:** 10.1007/s43390-024-00990-0

**Published:** 2024-10-23

**Authors:** Stefan Lang, Moritz Jokeit, Ji Hyun Kim, Lukas Urbanschitz, Luca Fisler, Carlos Torrez, Frédéric Cornaz, Jess G. Snedeker, Mazda Farshad, Jonas Widmer

**Affiliations:** 1 Spine Biomechanics, Department of Orthopedics, Balgrist University Hospital, University of Zurich, Zurich, Switzerland; 2 Department of Orthopedics, Balgrist University Hospital, University of Zurich, Zurich, Switzerland; 3 Institute for Biomechanics, ETH Zurich, Zurich, Switzerland

**Keywords:** Deep learning, Spinopelvic parameters, Cobb angle, Landmark detection, Automated parameter prediction

## Abstract

**Introduction:**

Accurate landmark detection is essential for precise analysis of anatomical structures, supporting diagnosis, treatment planning, and monitoring in patients with spinal deformities. Conventional methods rely on laborious landmark identification by medical experts, which motivates automation. The proposed deep learning pipeline processes bi-planar radiographs to determine spinopelvic parameters and Cobb angles without manual supervision.

**Methods:**

The dataset used for training and evaluation consisted of 555 bi-planar radiographs from un-instrumented patients, which were manually annotated by medical professionals. The pipeline performed a pre-processing step to determine regions of interest, including the cervical spine, thoracolumbar spine, sacrum, and pelvis. For each ROI, a segmentation network was trained to identify vertebral bodies and pelvic landmarks. The U-Net architecture was trained on 455 bi-planar radiographs using binary cross-entropy loss. The post-processing algorithm determined spinal alignment and angular parameters based on the segmentation output. We evaluated the pipeline on a test set of 100 previously unseen bi-planar radiographs, using the mean absolute difference between annotated and predicted landmarks as the performance metric. The spinopelvic parameter predictions of the pipeline were compared to the measurements of two experienced medical professionals using intraclass correlation coefficient (ICC) and mean absolute deviation (MAD).

**Results:**

The pipeline was able to successfully predict the Cobb angles in 61% of all test cases and achieved mean absolute differences of 3.3° (3.6°) and averaged ICC of 0.88. For thoracic kyphosis, lumbar lordosis, sagittal vertical axis, sacral slope, pelvic tilt, and pelvic incidence, the pipeline produced reasonable outputs in 69%, 58%, 86%, 85%, 84%, and 84% of the cases. The MAD was 5.6° (7.8°), 4.7° (4.3°), 2.8 mm (3.0 mm), 4.5° (7.2°), 1.8° (1.8°), and 5.3° (7.7°), while the ICC was measured at 0.69, 0.82, 0.99, 0.61, 0.96, and 0.70, respectively.

**Conclusion:**

Despite limitations in patients with severe pathologies and high BMI, the pipeline automatically predicted coronal and sagittal spinopelvic parameters, which has the potential to simplify clinical routines and large-scale retrospective data analysis.

## Introduction

In recent years, the analysis of spinal alignment has gained paramount importance in the diagnosis, treatment, and monitoring of spinal diseases, particularly in conditions, such as adolescent idiopathic scoliosis (AIS) [1] and adult spinal deformity (ASD) [2]. These conditions, characterized by complex spinal curvatures and deformities, present significant challenges in clinical management and treatment planning [3, 4]. In the realm of spinal surgery, the primary objective is often to restore spinal alignment to as close to normal as possible. Surgery that aims to restore spinal alignment can profoundly impact a patient’s quality of life as it is critical for reducing pain, improving function, and halting the progression of deformity. Understanding the intricate relationships between various spinopelvic alignment parameters is essential for uncovering the underlying causes of spinal deformities and optimizing treatment strategies. In the past decades, spinopelvic parameters have gained more attention as they have been associated with lower back pain [5, 6]. Parameters such as lumbar lordosis or sacral slope are used more frequently to restore spinal balance by surgical treatment [7]. Further, they appear to be predictive for complications in spinal fusion surgery [8].

The advent of modern imaging techniques has revolutionized diagnosis and monitoring of spinal diseases. However, the conventional approach to analyzing spinal alignment relies heavily on manual identification and measurement of anatomical landmarks on radiographic images. This process is time-consuming, subject to inter- and intra-observer variabilities, and often slows down clinical workflows.

Several approaches have been introduced to automatically extract position data and calculate spinal parameters, e.g., the Cobb angle in scoliosis patients [9] or lumbar parameters, such as sacral slope and pelvic tilt [10]. Thus far, no approach has established a pipeline for the automated and combined acquisition of the spine’s and pelvis positional data and calculation of the spinopelvic parameters. Besides, the used datasets to test the model are often too small or lack diversity to represent spinal pathologies in clinical situations [10].

**Fig. 1:**
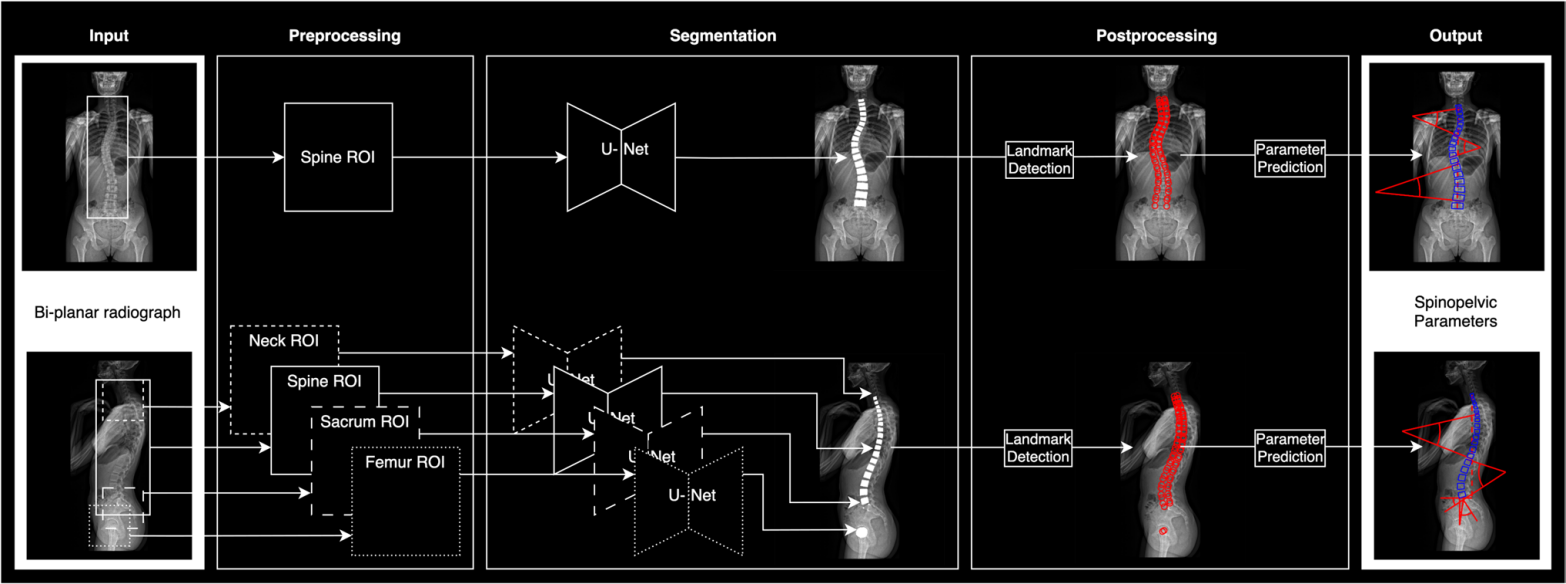
Overview of fully automatic pipeline for spinopelvic parameter prediction

In this context, we introduce a novel deep learning pipeline designed to process bi-planar radiographs for the automated determination of spinopelvic parameters without the need for manual intervention (Fig. 1). This study assessed and validated the extraction of the following spinopelvic parameters from biplanar radiographs: Cobb angle (Cobb°), thoracic kyphosis (THK°), lumbar lordosis (LL°), sagittal vertical axis (SVA), sacral slope (SS°), pelvic tilt (PT°), and pelvic incidence (PI°). By reducing reliance on manual measurements, the proposed approach aims to mitigate the subjective elements inherent in traditional methods and provide a more standardized, efficient, and reproducible framework for spinal deformity analysis.

## Methods

### Subjects

After approval of the institutional review board and the local ethics commission, a medical professional selected and anonymized bi-planar radiographs from the institutional imaging database. Anterior–posterior and lateral radiographs were acquired with the EOS^®^ Imaging System between October 2011 and November 2020. Patients with the inability to stand and spinal instrumentation were excluded. The selection comprised 555 bi-planar radiographs from 393 patients between 8 and 92 years (mean 42.2 ± 24.3 years).

### Data acquisition

On all anterior–posterior and lateral radiographs, 232 patient-specific anatomical landmarks were annotated by clinicians and medical students using a custom annotation tool written in MATLAB (Version 9.9 R2020b, The MathWorks, Inc.). The annotations were used to create binary segmentation masks of the thoracolumbar spine (C7–L5), the sacral endplate and the femur heads. The separate segmentations of C7 and the sacral endplate provided upper and lower boundaries for the segmentation of all vertebrae in between. The rectangular vertebrae masks were generated using the four vertebra corner coordinates. The sacral endplate masks consisted of a circular region on the anterior and posterior sacral endplate corner point respectively. The mask of the femoral head was created by fitting a circle through the three annotated points on it. Of 555 radiographs, 100 were randomly chosen and retained as the hold-out data (test set), while the remaining 455 image pairs were used for training the deep learning pipeline (training set).

### Pipeline

As presented in Fig. 1, the pipeline was divided into three subtasks: preprocessing, segmentation, and postprocessing. To simplify the segmentation task, input images and masks were processed in MATLAB (Version 9.9 R2020b, The MathWorks, Inc.) and automatically cropped into four regions of interest (ROI): neck, spine, sacrum, and femur. The desired cropping window was determined by creating histograms of projected intensity through summing up pixel values in the horizontal and vertical directions [9]. Horizontally, the head, shoulder girdle, and pelvis height showed prominent maxima, while the neck and abdomen level showed identifiable minima. In the vertical direction, a single peak was identified. Local maxima and minima, which exceeded a normed intensity threshold of 0.6, were used to crop out the four ROIs on images and masks. Downsizing and normalization completed the preprocessing step.

**Fig. 2:**
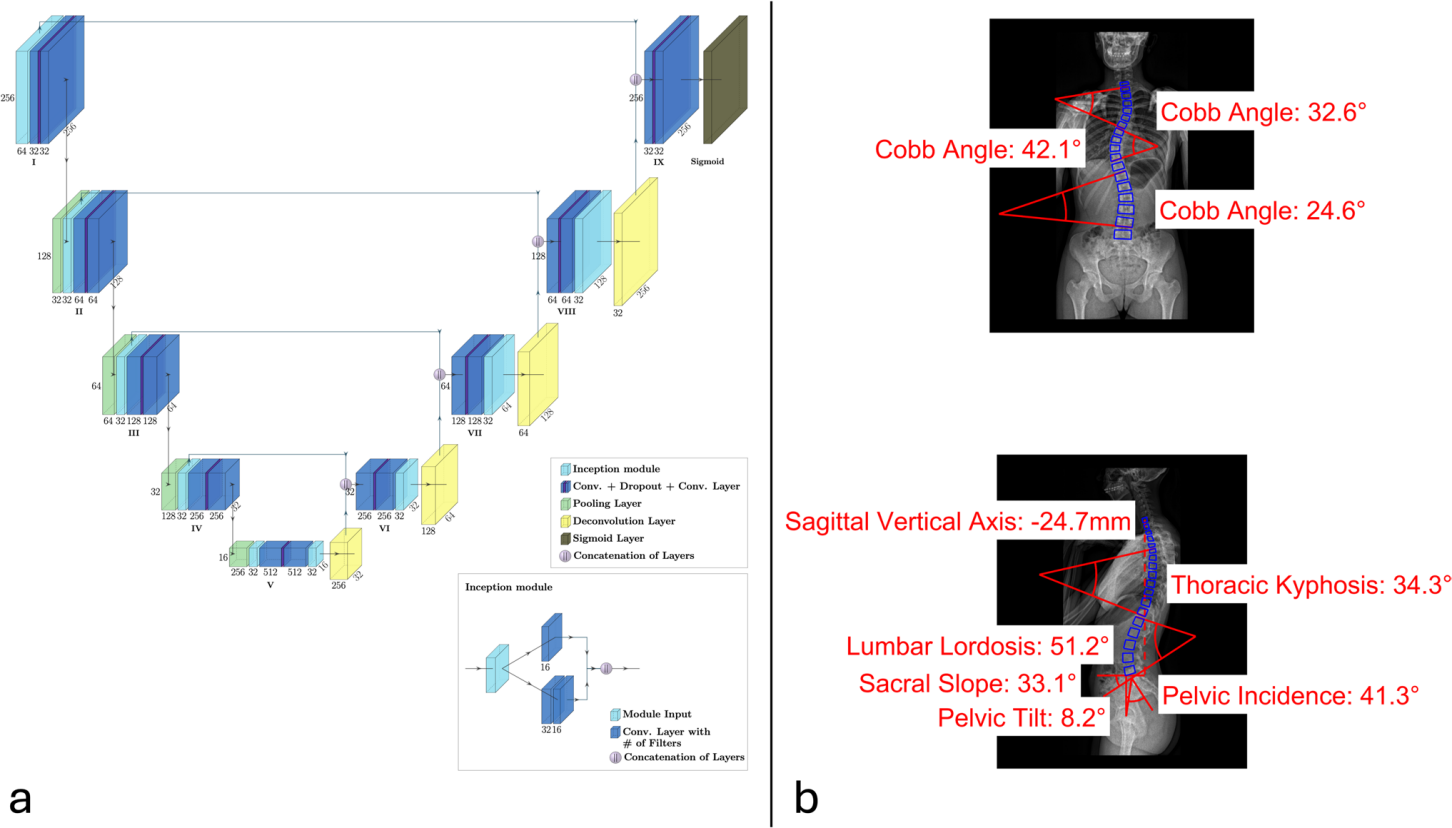
Modified U-Net architecture with inception modules of the segmentation network (**a**) and exemplary output predictions of Cobb angles and sagittal spinopelvic parameters (**b**)

For each ROI, a modified U-Net with inception modules [11, 12] was implemented using TensorFlow library for Python. The detailed architecture of the segmentation network is depicted in Fig. 2. The training for each individual network was conducted on a NVIDIA RTX A6000 GPU by minimizing the binary cross-entropy loss using the Adam optimizer [13] with an initial learning rate of 0.0005. The network weights were initialized using He-normal initialization scheme [14] and all networks were trained for 500 epochs with a batch size of 20. The training set was further subdivided with ratio of 85:15, leaving 387 samples for training and 68 samples for hyperparameter tuning (validation set).

The postprocessing was conducted with a custom MATLAB program. For each ROI, the output of the segmentation network was turned into binary segmentation masks using a threshold of 0.7. Inversion of the preprocessing steps ensured a correct superimposition of the predicted mask onto the anterior–posterior and lateral input radiographs. First, the neck ROI used to localize the C7 vertebral body was merged into the spine ROI to rectify incorrect predictions of C7 within the spinal ROI. Similarly, the segmentation of the sacral endplate points was used to correct vertebra segmentations caudally. Then, an algorithm was employed to identify the corner points in the rectangular segmentations of the vertebral bodies. The midpoint of the sacral endplate was determined by averaging the anterior and posterior sacral endplate point predictions. The program derived the midpoints of the anterior and posterior femoral heads and transferred all landmarks into input image coordinates. Finally, the corner points of the vertebral bodies, the sacral endplate points, and the centers of the femoral head were used to calculate the spinopelvic parameters. An example prediction of the calculated parameters can be seen in Fig. 2.

### Evaluation of clinical parameter predictions

The pipeline was evaluated separately for the preprocessing, segmentation, and postprocessing subtasks on a test set of 100 previously unseen radiographs. The preprocessing step was evaluated based on the number of radiographs that were processed correctly. The preprocessing was considered as successful, if the ground truth completely lay inside the processed image and was not cut off. The segmentation quality was evaluated using the F1 score (or Dice coefficient), while the landmark predictions were compared to the corresponding manual annotations using the mean absolute deviation (MAD). The segmentation was considered as reasonable, if the spine ROI prediction consisted of at least 18 vertebral bodies and a prediction for each ROI was made. To evaluate the accuracy of the final parameter predictions, a blinded medical expert measured the spinopelvic parameters in the test set using mediCAD Software (Classic Version 6.0.0.9, mediCAD Hectec GmbH). The inter-rater reliability among three different medical experts was assessed using the intraclass correlation coefficient (ICC) based on a subset of 10 bi-planar radiographs. To assess the intra-rater reliability, one expert repeated three measurements at three-day intervals using the same subset and metric. For both intra- and inter-rater reliabilities, the ICC type was chosen as: two-way mixed effects, single measurement, and absolute agreement. The reliability of the results was classified into four grades according to the value of the ICC: excellent (0.9–1.0), good (0.75–0.9), moderate (0.5–0.75), and poor (< 0.5) [15].

## Results

The testing dataset comprised 58 scoliosis patients in total. The distribution according to Cobb [16] and the classification of thoracic kyphosis following the Lenke classification [17] is shown in Table 1.

**Table 1:** Mean and standard deviation (SD) of the Cobb° and THK° from T5 to T12 in the test data [17]

Cobb (θ°)	θ < 10°	10° ≤ θ < 25°	25° ≤ θ < 45°	θ ≥ 45°	Total
Number of patients	42	40	14	4	100
Cobb° (SD)	0°	14.8° (3.9°)	34.8° (5.9°)	56.9° (6.5°)	22.6° (13.6°)

THK (φ°)	φ < 10°	10° ≤ φ < 40°	φ ≥ 40°	Total
Number of patients	3	39	28	70
THK° (SD)	6.6° (3.2°)	27.6° (8.1°)	50.8° (12.0°)	33.5° (14.7°)

According to the guidelines from [15], all three raters showed good agreement (0.75–0.9) for the Cobb angles (Table 2). In case of LL, SS, and PT, the raters achieved an excellent agreement (> 0.9) but only good and moderate (0.5–0.75) ICC values for SVA and THK, respectively. The inter-rater error averaged over all raters for the Cobb angles was 6.2° (8.9°) and varied between 2.2° (3.9°) and 7.1° (7.7°) for THK, LL, SS, and PT. The mean absolute distance for SVA was 6.2 mm (10.0 mm). The intra-rater reliability for the Cobb angles was excellent but moderate for LL, SS, and PT. The intra-rater agreement was good and excellent for SVA and THK, respectively.

**Table 2:** Intraclass coefficient (ICC) with 95% confidence interval (CI) for inter-rater reliability and intra-rater reliability as well as mean absolute deviation (MAD) for coronal and sagittal spinopelvic parameters on a subset of 10 bi-planar radiographs

Parameter	Cobb 1	Cobb 2	Cobb 3	Total	THK	LL	SVA	SS	PT
Inter-rater reliability									
ICC (inter-rater)	0.81	0.86	0.73	0.82	0.59	0.91	0.87	0.92	0.91
95% CI	0.61–0.83	0.64–0.96	0.06–0.99	0.68–0.82	0.22–0.86	0.73–0.98	0.67–0.96	0.79–0.98	0.77–0.98
Intra-rater reliability									
ICC (intra-rater)	0.95	0.97	0.79	0.95	0.96	0.71	0.85	0.54	0.62
95% CI	0.90–0.95	0.93–0.99	0.36–0.97	0.91–0.95	0.93–0.98	0.18–0.90	0.72–0.93	0.27–0.76	0.38–0.81
Inter-rater error									
R1-R2 MAD (SD)	9.3° (13.1°)	5.3° (5.7°)	3.5° (8.6°)	8.3° (10.8°)	6.8° (6.9°)	4.8° (3.7°)	8.6 mm (12.0 mm)	3.4° (3.3°)	2.6° (5.0°)
R2-R3 MAD (SD)	8.7° (13.8°)	2.0° (2.4°)	3.9° (10.9°)	6.5° (11.9°)	5.3° (4.3°)	3.9° (2.1°)	1.9 mm (1.6 mm)	3.6° (2.7°)	1.7° (0.8°)
R3-R1 MAD (SD)	2.8° (1.7°)	4.1° (5.6°)	1.7° (3.0°)	3.9° (4.1°)	9.1° (10.3°)	5.1° (4.5°)	8.1 mm (11.3 mm)	4.2° (3.0°)	2.2° (4.3°)
Average MAD (SD)	6.8° (11.4°)	3.8° (5.0°)	3.0° (8.3°)	6.2° (8.9°)	7.1° (7.7°)	4.6° (3.6°)	6.2 mm (10.0 mm)	3.7° (3.1°)	2.2° (3.9°)

*R1* rater 1, *R2* rater 2, *R3* rater 3, *Cobb° 1* most caudal Cobb angle, *Cobb° 2* (if present): next cranial Cobb angle, *Cobb° 3* (if present): most cranial Cobb angle, *Total* mean over all Cobb angles, *THK* thoracic kyphosis, *LL* lumbar lordosis, *SVA* sagittal vertical axis, *SS* sacral slope, *PT* pelvic tilt, *PI* pelvic incidence

The pipeline’s processing performance is summarized in Table 3. The automated preprocessing of the images, i.e., the image cropping and resizing, successfully created correct ROIs in 533 out of 555 samples (96%). As the

**Table 3:** Preprocessing: number of correctly preprocessed samples for the neck, spine, sacral endplate, femoral head ROI and for the whole testing dataset. Segmentation: number of predicted regions and the corresponding F1-score of the testing dataset. Spinopelvic parameter prediction: Number of reasonable frontal and lateral predictions on the total testing dataset and the scoliosis patient subset

Pipeline’s processing performance											
ROI	Neck	Spine (front./lat.)		Sacrum (ant./post.)			Femur (ant./post.)			Total	
Preprocessing											
Correct preprocessing	552/555	548/555 / 538/555		554/555 / 554/555			553/555 / 552/555			533/555	
%	99.5%	98.7% / 96.9%		99.8% / 99.8%			99.6% / 99.5%			96.0%	
Segmentation											
Reasonable segmentation	96/100	61/100 / 69/100		92/100 / 89/100			100/100 / 98/100			45/100	
F1-score	0.73	0.86 / 0.86		0.49 / 0.52			0.83 / 0.76			−	
Spinopelvic parameter prediction											
Parameter		Cobb	THK		LL	SVA		SS	PT		PI
Reasonable prediction		61/100	69/100		58/100	86/100		85/100	84/100		84/100
Reasonable prediction on scoliosis patients		34/58									

pipeline successfully predicted all spinopelvic parameters for 45 out of 100 patient images, a reasonable segmentation was achieved in 45% of all cases. Table 3 shows the segmentation results and the F1 score for each ROI. The prediction of the spinopelvic parameters was only conducted for patients with complete segmentations (61 frontal and 45 frontal + lateral). Patients with any predictions from a region missing were excluded and not further processed. Predictions of the frontal parameters (predicted Cobb angles ≤ 10° in patients without scoliosis were also considered as reasonable) were made in 61 of 100 patients. 34 out of 58 (58.6%) predictions were successfully made in patients with scoliosis.

Table 4 shows the MAD and SD for a single corner point between the manual annotations and the predicted coordinates for the thoracic region (including C7), the lumbar region and both regions combined. One patient was excluded, as a foreign object on the radiograph led to a faulty segmentation. Further, Table 4 presents the error between the anterior and posterior sacral endplate point, respectively femoral head and between the midpoint of both anterior and posterior points (sacral endplate midpoint and hip axis midpoint).

**Table 4:** Mean absolute deviation (MAD) and standard deviation (SD) between the manually annotated spinopelvic landmarks and the predicted coordinates

Landmark detection: pipeline versus manual annotations				
Region	Frontal	Lateral	Sacral Endplate	Femoral Head
Thoracic MAD (SD)	3.9 mm (2.9 mm)	6.4 mm (6.3 mm)		
Lumbar MAD (SD)	4.0 mm (3.4 mm)	3.9 mm (6.2 mm)		
Total MAD (SD)	4.0 mm (2.8 mm)	6.8 mm (6.4 mm)		
Anterior MAD (SD)			3.2 mm (5.1 mm)	4.7 mm (4.2 mm)
Posterior MAD (SD)			2.6 mm (4.4 mm)	4.4 mm (3.3 mm)
Midpoint MAD (SD)			2.4 mm (4.2 mm)	3.2 mm (2.5 mm)

**Fig. 3:**
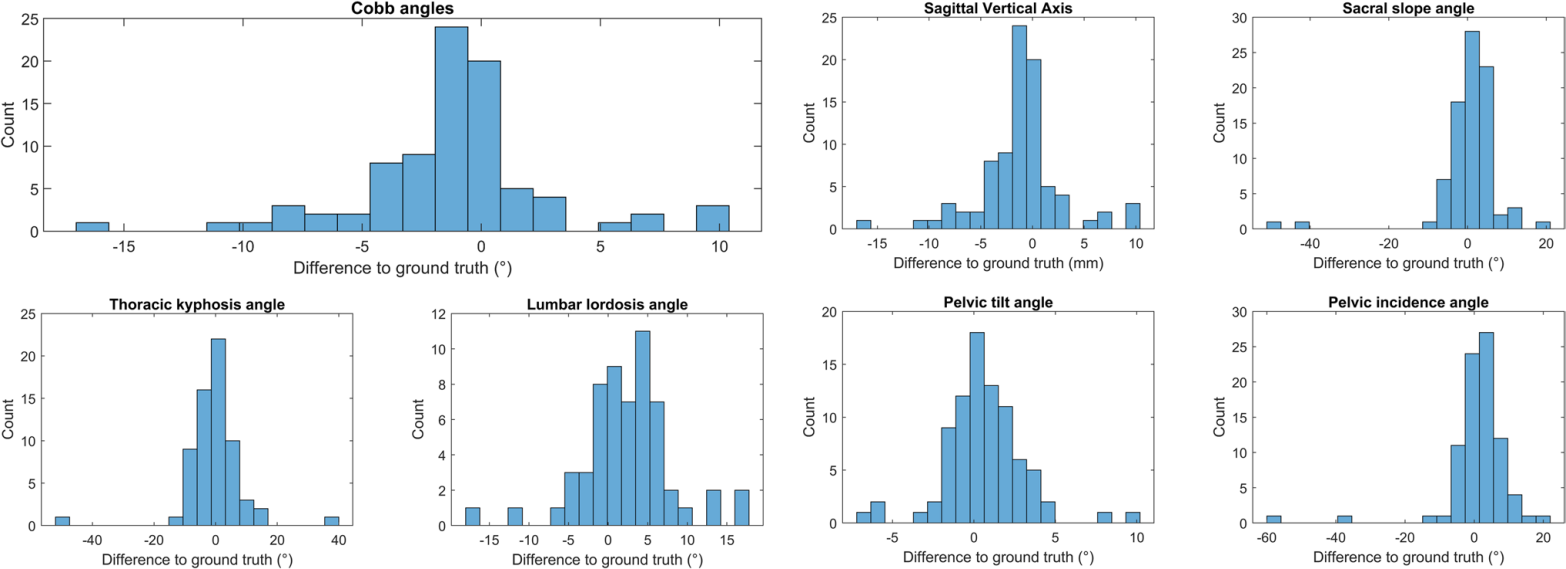
Error distributions for Cobb angles and lateral spinopelvic parameters in comparison to the expert measurements for all successfully processed cases from the test set

The agreement between the spinopelvic parameter predictions of the pipeline and the rater is presented in Table 5. For the Cobb angles, the pipeline showed good to excellent agreement with the clinician. From the sagittal parameters, THK and SS exhibited moderate agreement while LL was in good agreement and SVA and PT in excellent agreement with the rater. This is also reflected in the inter-rater error, which averaged 3.3° (3.6°) for the Cobb angles and varied between 1.8° (1.8°) and 5.6° (7.8°) for the sagittal parameters. The mean absolute distance for SVA was 2.8 mm (3.0 mm). The histograms in Fig. 3 show the error distribution for all successful spinopelvic parameter predictions.

**Table 5:** ICC with 95% confidence interval (95% CI) and mean absolute deviation (MAD) between the expert measurements and the predicted parameters for all successful predictions (61 frontal and 45 frontal + lateral)

Spinopelvic parameter prediction: pipeline versus rater 1									
Parameter	Cobb 1	Cobb 2	Cobb 3	Cobb average	THK	LL	SVA	SS	PT
ICC	0.86	0.94	0.94	0.88	0.69	0.82	0.99	0.61	0.96
95% CI	0.73–0.86	0.84–0.98	0.67–0.99	0.81–0.93	0.55–0.80	0.67–0.90	0.99–1.00	0.45–0.72	0.93–0.97
MAD (SD)	3.7° (4.3°)	2.9° (1.8°)	2.0° (1.6°)	3.3° (3.6°)	5.6° (7.8°)	4.7° (4.3°)	2.8 mm (3.0 mm)	4.5° (7.2°)	1.8° (1.8°)

## Discussion

The introduction of deep learning methods in the analysis of bi-planar radiographs represents a significant advancement in the field of spinal deformity diagnosis and monitoring. The proposed deep learning pipeline demonstrated substantial potential in automating the process of determining spinopelvic parameters and Cobb angles, traditionally reliant on time-consuming manual identification by medical experts. In contrast to comparable approaches, it combines the prediction for both planes and is not limited to a certain spinal region [18–20] or age group [9]. In this study, the Cobb angle’s predictive accuracy showed a mean absolute deviation (MAD) of 3.3°, which is slightly higher than the 2.4° deviation found in a recent pediatric study [9]. Furthermore, 80% of the predictions were within ± 5° of the true values, which aligns with the expected range of human observer error [21, 22]. The study found that most predicted values for sagittal spinopelvic parameters, including THK, LL, SS, PT, and PI, were within ± 5° of the ground truth. Additionally, 85% of SVA measurements were within the acceptable error margin of ± 5.7 mm [21]. Recent studies have reported similar results, which are summarized in Table 6. It is worth noting that only a few extreme outliers were observed in lateral parameters. THK° errors were mainly due to incorrect vertebrae segmentation, while SS° discrepancies resulted from inconsistent sacral endplate level predictions. The predicted angles and SVA metrics closely matched those of recent studies, with SVA slightly exceeding approach [10].

**Table 6:** Pipeline’s achieved mean absolute deviation (MAD) in comparison with other recent approaches [9, 10, 18–20, 26, 27]

Comparison with state-of-the-art							
Parameter	Cobb	THK	LL	SVA	SS	PT	PI
Imran et al. [9]	2.4°	–	–	–	–	–	–
Wu et al. [23]	4.0°	–	–	–	–	–	–
Schwartz et al. [18]	–	–	–	–	2.1°	4.8°	4.6°
Korez et al. [19]	–	–	–	–	5.0°	2.7°	5.5°
Cina et al. [20]	–	–	1.84°^1^	–	1.98°	–	–
Galbusera et al. [24]	5.1°	7.6°^2^	0.8°^1^	–	6.2°	0.7°	6.9°
Yeh et al. [10]	–	5.4°	5.1°	1.9 mm	3.5°	1.1°	3.8°
**Presented pipeline**	**3.3°**	**5.6°**	**4.7°**	**2.8 mm**	**4.5°**	**1.8°**	**5.3°**

The results of the presented pipeline are highlighted in bold to aid the comparison with other approaches

^1^L1-L5 lumbar lordosis

^2^T4-T12 thoracic kyphosis

The main limitation of the presented pipeline was that it only fully processed 45% of the unseen test data. The robustness on the coronal parameters was slightly superior with 61% processed radiographs. In rare cases, deceptive peaks in the vertical intensity distributions occurred due to prostheses, implants, severe obesity, pronounced thoracic kyphosis, or hand placement, which caused the histogram-based ROI selection to fail. Potential improvements for preprocessing include fine-tuning intensity thresholds or choosing more generous cropping windows. However, as preprocessing proved to be very robust (96% processing rate), the pipeline’s failure was mainly attributed to the segmentation task, which was crucial for correct post-processing and parameter prediction. The F1 scores for the segmentation of the ROIs ranged from 0.49 to 0.86. The spine ROI achieved the highest scores (0.86 for both frontal and lateral), while the sacral endplate (0.49 anterior and 0.52 posterior circle) and femoral head (0.83 anterior and 0.76 posterior) achieved lower scores. Previous studies have reported higher F1 scores, such as 0.92 in [9], and 0.93 in [23] for the frontal spine and 0.94 in [23], for the segmentation of the lateral spine. In radiographic analysis, segmentation accuracy is often diminished in obese or osteoporotic patients due to a reduction in bone signal. The thoracic region is particularly prone to segmentation errors due to the superimposition of adjacent anatomical structures like humerus, scapula, and ribs. While there is no existing literature to benchmark the segmentation F1-scores for the sacral endplate or femoral head, their scores are notably lower than those for the spine region, with the anterior and posterior femoral head at 0.83 and 0.76, and the anterior and posterior sacral endplate point at 0.49 and 0.52, respectively. However, these lower F1 scores should not be interpreted as indicative of poor segmentation quality. As Rohlfing pointed out [24], F1 scores cannot be directly compared across different segmentation targets due to size variance. As the segmentation output threshold was set to 0.7, further adjustments could optimize segmentations and output predictions. Additionally, sophisticated postprocessing could allow parameter prediction from qualitatively less accurate segmentations.

Despite the large age range of the patients, the pipeline was able to learn the correct segmentation and annotation of anatomical features. Fine-tuning the model for individual age classes may improve accuracy but minimizes the generalizability of the model. Manual annotation of anatomical landmarks introduces some bias to the training data. However, using multiple annotators and a programmatic annotation protocol reduced the risk of single source bias. Like other data-driven approaches, the limited availability of annotated medical imaging data hinders the training and refinement of deep learning models, which may affect their accuracy and generalizability. Given the complexity of the segmentation task, the current data selection may be underpowered compared to [10] and [25] who used datasets of over 2000 radiographs. Further, metallic implants caused failures in the intensity-based pre-processing and segmentation. It is important to note that patients with spinal instrumentation must also be considered for effective automated analysis in diverse patient populations. For future works, the extension of the dataset is mandatory to increase overall robustness and accuracy of landmark identification and parameter predictions.

## Conclusion

Overall, the interclass correlation coefficient (ICC) between expert measurements and predicted parameters was excellent for Cobb° 2, Cobb° 3, sagittal vertical axis (SVA), and pelvic tilt (PT°). Good values were achieved for Cobb° 1, total Cobb°, and lumbar lordosis (LL°), while thoracic kyphosis (THK°), sacral slope (SS°), and pelvic incidence (PI°) achieved only moderate results. Although segmentation of sacral endplate points and femoral heads showed promising results, vertebra segmentation requires enhancement, particularly in the thoracic region. Despite the discussed challenges, the results demonstrate the feasibility of using deep learning to predict spinopelvic parameters from frontal and lateral radiographs. The pipeline achieved comparable results to other recent approaches and indicates promising avenues for reducing bias in clinical routines and facilitating extensive retrospective data analysis.

## Data Availability

The datasets generated during and/or analysed during the current study are not publicly available due to reasons of sensitivity. The code is available from the corresponding author upon reasonable request.
